# Surveillance of Soil-Transmitted Helminth Infection in Preschool Child Population: Do Changes in Behavior and Immunological Responses Affect Prevalence?

**DOI:** 10.3390/tropicalmed9020033

**Published:** 2024-01-30

**Authors:** Riyadi Adrizain, Monika Verena Nagari, Hadyana Sukandar, Afiat Berbudi, Djatnika Setiabudi, Budi Setiabudiawan

**Affiliations:** 1 Department of Child Health, Faculty of Medicine, Universitas Padjadjaran, Bandung 40161, Indonesiabudi.setiabudiawan@unpad.ac.id (B.S.); 2 Faculty of Medicine, Universitas Padjadjaran, Bandung 40161, Indonesia; 3 Department of Public Health, Faculty of Medicine, Universitas Padjadjaran, Bandung 40161, Indonesia; 4 Department of Biomedical Science, Faculty of Medicine, Universitas Padjadjaran, Bandung 40161, Indonesia; a.berbudi@unpad.ac.id; 5 Faculty of Medicine, President University, Bekasi 17550, Indonesia

**Keywords:** STH infection, stunting, 25(OH)D, IL-5, IL-13

## Abstract

Soil-transmitted helminths (STHs) persist as a significant global public health issue among neglected tropical diseases (NTDs), particularly in children. STH infection can induce immune responses that affect the course of the disease; if treatment fails, chronic infection can lead to stunting, especially among children aged 24–59 months, which is a vulnerable period for growth and development. We conducted a correlational, cross-sectional data collection study to evaluate the characteristics and association of 25(OH)D, interleukin-5 (IL-5), and interleukin-13 (IL-13) with the prevalence of STH infection in children aged 24–59 months in Bandung District, Indonesia, in October 2019–January 2023. We recruited 694 subjects (401 stunted and 293 normal-height children). The prevalence of STH infection among the stunted and normal-height groups was 5.7% (95% CI: 3.85–8.46%) and 3.4% (95% CI; 1.86–6.17%) (*p* = 0.156), respectively. The probability of the prevalence of STH infection in children with levels of 25(OH)D, IL-5, and IL-13 below the cut-off point was 6,93 to 16.71 times higher. We found a relationship between IL-5, IL-13, and environmental factors and the prevalence of STH infection in stunted children.

## 1. Introduction

The neglected tropical diseases (NTDs) category of tropical illnesses is the focus of worldwide activities aimed at disease control and elimination. Soil-transmitted helminths (STHs) are one of the 20 NTDs that remain a global public health problem [1]. In 2015, the World Health Organization (WHO) reported that more than 24% of the world’s population was infected with helminths, 60% of which were children [2]. Parasitological surveys conducted in Indonesia in the 1980s and 1990s estimated that the prevalence of *Ascaris lumbricoides*, *Trichuris trichiura*, and hookworm ranged between 14 and 90%, 1 and 91%, and 21 and 89%, respectively. This estimate, which is based on data that are more than two decades old, needs updating [3].

The WASH (Water, Sanitation, and Hygiene) program launched by the WHO focuses on health, livelihood, school attendance, and dignity and helps create resilient communities living in a healthy environment [4]. The findings of a study conducted in Indonesia in 2016 on children under five years old identified a relationship between the prevalence of STH infection and the cleanliness of water sources, the presence of fecal disposal facilities, the conditions of wastewater disposal facilities, and the type of house floor [5]. Safe and appropriate WASH is critical for the prevention of NTDs such as trachoma, STH, and schistosomiasis. Evidence suggests that properly managed drinking water and sanitation, such as regulated piped water and sewer connections with wastewater treatment, can substantially benefit health by lowering fatalities due to diarrheal disease [4].

The Worm Eradication Program has been implemented in Indonesia, following the regulation of Health Minister No. 15/2017, which states that health promotion, helminth surveillance, control of risk factors, patient management, and mass drug administration such as single-dose albendazole or mebendazole should be implemented to decrease the number of infections [6].

Parasite infection can cause digestive tract problems during the process of absorbing and digesting foods. Chronic inflammation can occur, as well as nutritional loss owing to direct nutrient absorption by parasites or the use of calories to support the immune system to fight the parasites [7,8]. Malnutrition, iron deficiency anemia, vitamin A deficiency, and vitamin D deficiency are caused by helminth infection. Additionally, low socioeconomic status and limited access to health services are associated with the prevalence of STH infection and micronutrient deficiencies [9].

Vitamin D plays a role in malignancy, allergies, and infections. Vitamin D enhances the antiparasite response in the innate immune system and the anti-inflammatory effects in the adaptive immune system. Research conducted over the last twenty-five years revealed that 1.25(OH)_2_D_3_ also has strong immune-modulating effects [10].

The role of the adaptive immune system in parasitic infections is crucial in the elimination and repair of postinfectious tissue. Vitamin D regulates inflammation in infected individuals through the effects of T cells. Vitamin D also appears to suppress the proliferation and production of T-helper 1 cytokines while promoting the proliferation and production of T-helper 2 cells and the production of regulatory T cells. Given the inflammatory effects of TNF-α, INF-γ, and other Th1 cytokines, optimizing vitamin D status, especially preventing deficiency, can prevent the dysregulation and circulation of more proinflammatory cytokines in infected individuals, which can play a role in decreasing the severity of the disease [11,12,13].

The immune response is time-related, with increased levels of IgE, IL-5, and IL-13 found in older individuals experiencing STH infection and reduced levels of Th1-related cytokines. This evidence supports the role of the controlled suppression of the STH immune response in reducing the burden of worms, inhibiting the formation of the larval epithelium, activating the Th2 response barrier, and restoring the integrity of the mucosal barrier and the diversity of the microbiome. The roles of IL-5, IL-13, and memory Th2 cells are crucial; if this function is disrupted, the ability to expel STHs is weakened, leading to damage to the mucosal barrier. Tissue repair does not occur [14].

In this study, we aimed to evaluate the prevalence of STH infection and its relationship with behavior, environmental factors, and immunology responses.

## 2. Materials and Methods

### 2.1. Study Design

We conducted a cross-sectional, correlation study of the collected data to evaluate the characteristics and association of 25(OH)D, IL-5, and IL-13 with the prevalence of STH infection in children aged 24–59 months.

### 2.2. Study Site and Population

From October 2019 to January 2023, a study was undertaken in Bandung, West Java, Indonesia. This study’s object was the population of children aged 24 to 59 months residing in the area. During data collection, children who moved away from the study area, had a severe congenital defect or syndrome, took deworming medications in the previous 6 months, took part in a mass drug administration campaign for STH in the previous 1 month, or took vitamin D supplements were excluded from the study.

### 2.3. Study Size and Sampling

The sample size was calculated to determine the prevalence of helminthiasis in stunted children using the formula n = p$\left(1-p\right){\left(\frac{Z\alpha }{d}\right)}^{2}$, where n is the sample size, p is the estimate of STH infection prevalence, Zα is the standard deviation obtained from the standard normal distribution table for the selected confidence level, and d is the margin of error.

The assumption of a 95% confidence level (Zα = 1.96) was used to estimate the prevalence (p) of helminthiasis as 0.20 with a 0.05 margin of error (d). To obtain a sufficient sample size to evaluate the association of 25(OH)D, IL-5, and IL-13, we used the following formula to test the difference between two means:

$$n=\frac{2S^{2}{\left(Z\alpha +Z\beta \right)}^{2}}{d^{2}}$$

where n is the sample size, and S is the combined standard deviation obtained from

$$S=\sqrt{\frac{\left(n_{1}-1\right)S_{1}^{2}+\left(n_{2}-1\right)S_{2}^{2}}{\left(n_{1}+n_{2}-2\right)}}$$

**Figure 1:**
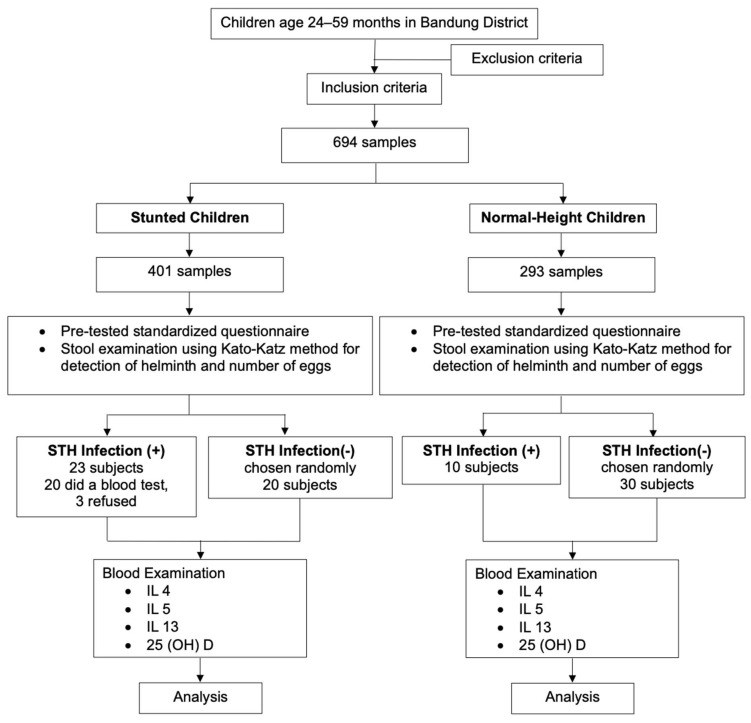
Flow chart of study participant selection process.

Zα+Zβ = Z is the deviation value obtained from the standard normal distribution table for the selected α significance level for the power test 1-β; d is the magnitude of the difference in the means of the variables studied in the two groups (children with and without STH infection). Using a statistical significance of 5% and a power test of 80%, the final minimum sample size was therefore calculated as 246. The minimum number of samples needed for a case–control study was 20 for stunted children with confirmed STH infection for the control group, and we needed 20 stunted children without STH infection, 10 normal-height children with STH infection, and 30 normal-height children without STH infection. The participants were recruited using a multistage random sampling technique. First, of the 31 subdistricts, 13 were selected. Second, of the 13 subdistricts, 39 villages were selected. In the 39 villages, we chose 29 primary healthcare centers. Finally, a simple random sampling technique was used to select children aged 24–59 months. Figure 1 illustrates the participant selection process in the study.

### 2.4. Variables Measurement

*Stunting.* Stunting was defined as a height-for-age Z score below −2 in the World Health Organization (WHO) growth reference standard.

*25(OH)D level.* The 25(OH)D level was measured as the concentration of the inactive form of vitamin D, which is the result of the metabolism of vitamin D2 and D3 by the 25-hydroxylase enzyme found in the liver. The levels were quantified using the ELISA method. The 25(OH)D levels were classified according to the Endocrine Society as deficiency (<20 ng/mL), insufficiency (21–29 ng/mL), or sufficiency (≥30 ng/mL).

*IL-5 level.* The IL-5 concentration in serum units was measured using the ELISA method.

*IL-13 level.* The IL-13 concentration in serum units was measured using the ELISA method.

*STH Infection.* STH infection was defined as positive when worm eggs (*A. lumbricoides*, *Trichuris trichiura*, and the hookworms) were found in stool samples. According to the WHO, mild-grade infections of *A. lumbricoides* range from 1 to 4999 eggs per gram of stool, moderate-grade infections range from 5000 to 49,999 eggs per gram of stool, and severe-grade infections involve more than 50,000 eggs per gram of stool [15].

The collected fecal samples were placed in sterile specimen cups. The serum levels of 25(OH)D, IL-5, and IL-13 of the subjects were measured using peripheral venous blood samples.

The Kato–Katz technique using the Serenity^®^ kit was used to detect common helminth species such as *Ascaris lumbricoides*, *Trichuris trichiura*, and hookworm. Euroimmune^®^ and Elabscience^®^ kits were used to determine the 25(OH)D, IL-5, and IL-13 levels in the blood samples; three slides prepared from each sample were analyzed for each patient using the Kato–Katz technique.

*Height and Weight.* Children who were at least 2 years old and capable of standing were weighed by themselves using a calibrated digital scale on a flat and solid surface. The scale was accurate to 0.1 kg. Height was assessed as maximum vertical body length; body measurements were recorded for children ≥2 years old who could stand without assistance. A stadiometer with an adjustable headpiece and a fixed vertical backboard was used to measure height. The stadiometer was placed on level ground. Subjects stood upright on a backboard with evenly distributed body weight and both feet flat on the platform. The back of the head, shoulder blades, buttocks, and heels touched the back board, aligning the head in the Frankfort horizontal plane. The measurement results were read in a position parallel to the meter, which was accurate to 0.1 cm.

### 2.5. Data Management and Analysis

The sociodemographic data of the subjects and anthropometric measures were obtained using a pretested standardized questionnaire. The sample collection was carried out by qualified field workers who had been standardized and received training directly or through audiovisual media, with supervision during the sample collection. The collected samples were sent to Dr. Hasan Sadikin Bandung General Hospital using standardized transport media within 30–60 min. The blood and fecal samples were then analyzed in the laboratory within 15 min.

The data were verified, coded, and entered into IBM SPSS v.29. Statistical analysis was performed using the chi-square, Fisher’s exact, Kruskal–Wallis, F, Mann–Whitney, unpaired T, Spearman correlation, and logistic double regression tests. The characteristics of the subjects were descriptively analyzed.

## 3. Results

### 3.1. Participant Characteristics

Between October 2019 and January 2023, 694 children were included in this study, comprising 33 (4.76%) children with and 661 (95.24%) children without STH infection. The characteristics of the participants are summarized in Table 1 The body height, mother’s occupation, father’s occupation, mother’s education, and nutritional status did not differ between the groups.

In the STH-positive group, only 2.6% resided more than 10 m away from a water tank. On the contrary, in the STH-negative group, the majority (97.4%) lived more than 10 m away, suggesting that the distance from water tanks is a significant risk factor for STH infection (*p* = 0.002). The proportion of children with STH infection was higher if the distance between water sources and the toilet was less than 10 m, in those who sourced their water from an electric water pump, and in those who had dirty toenails. Most of the cases were found in children whose fathers had a lower education level, and a significant difference was found with a prevalence of 12.5% (*p* < 0.001) (Table 1).

### 3.2. Comparison of the Severity Levels of STH Infection in Normal Children and Stunted Children Based on Etiology

The difference in severity levels between normal children and stunted children was examined in thirty-one subjects, comprising nine normal children and twenty-two stunted children. Based on the average eggs in each group, a statistical test was performed to assess the differences in severity levels between the two groups.

Most of the species that we found were *Ascaris lumbricoides*, but we also discovered other species in one sample infected with a mild *Trichuris trichiura* (146 epg) infection and one sample with a mild *Necator americanus* infection (235 epg). However, we excluded them from further investigation into immune responses to standardize the data, as their transmission dynamics differ. Based on Table 2, among the nine normal children, five were found to have mild infection, three had moderate infection, and one had a severe infection, and among the twenty-two stunted children, fifteen had mild infections, four had moderate infections, and three had severe infections. The average egg count in the mild infection group was 1.795 epg for normal children vs. 1.387 epg for stunted children. In the moderate infection group, the average egg count was 31.100 epg for normal children vs. 20.300 epg for stunted children. For the severe infection group, the average egg count was 172.250 epg for normal children vs. 60.800 epg for stunted children. However, there were no significant differences in the severity levels based on egg count between the groups of normal and stunted children (*p* > 0.05).

### 3.3. Correlation of 25(OH)D, IL-5, and IL-13 Levels with STH Infection

The 25(OH)D, IL-4, IL-5, and IL-13 levels in 80 subjects were used to divide them into a stunted group with STH infection (group A), a stunted group without STH infection (group B), a normal-height group with STH infection (group C), and a normal-height group without STH infection (group D). Table 2 shows a comparison of 25(OH)D, IL-5, and IL-13 levels from each group.

We found no significant differences in the 25(OH)D levels between groups. A significant difference in the IL-5 level was found between the groups of stunted children with and without STH infection (*p* = 0.006). The IL-13 levels were not significantly different between the group of stunted children with STH infection and the group of normal-height children without STH infection (*p* = 0.010) and in stunted children with STH infection compared with normal-height children without STH infection (*p* = 0.022). Because no significant differences were found in the normal-height groups with STH infection and without STH infection, further investigation was conducted only on the stunted groups.

A comparison of the characteristics of the stunted children with STH infection is shown in Table 3.

In the 40 stunted children, we found significant differences in the clean water sources (*p* = 0.020) and the distance from the water source to the toilet (*p* = 0.011).

The analysis was continued by looking for cut-off points for the studied variables to determine predictors of STH infection. The cut-off points for 25(OH)D, IL-5, and IL-13 levels are listed in Table 4. The AUROC results provided in the Table 5 are based on data on stunted children.

The cut-off point for each variable was obtained. Next, further calculations were conducted to assess the relationship between the cut-off points for 25(OH)D, IL-5, and IL-13 levels with the prevalence of STH infection in stunted children (Table 6).

Table 6 indicates the significant differences between all variables (25(OH)D, IL-5, and IL-13 levels) with the prevalence of STH infection. The odds ratio obtained was 6.93 for IL-5 and 16.71 for IL-13; for 25(OH)D, an odds ratio was not found.

Table 7 shows the results of multiple logistic regression analysis to determine the simultaneous effect of significant variables.

According to the findings of multivariable analysis, two variables, IL-5 and IL-13 levels, were simultaneously related to the prevalence of STH infection in stunted children. The R^2^ of 0.683 suggests that levels of IL-5 and IL-3 and an environmental variable (i.e., the distance from the water source to the toilet) explained 68.3% of stunted children with STH infection; the remaining 31.7% were influenced by other factors that were not considered.

## 4. Discussion

Our findings showed that the prevalence of STH infection was 4.7%, with no significant difference in prevalence between stunted and normal-height children. The prevalence of STH infection was much lower (<20%) in Bandung District than in Indonesia as a whole (24.6% to 26.9%). During this study, we found that children were more engaged in indoor activities; this was caused by the policies implemented at that time, which may have influenced children’s behavior, such as reduced outdoor play, studying from home, and parents working from home more. This resulted in a better impact on the well-being of children and lowered the risk of exposure to STH infection from environmental factors. In addition, given the lack of significant difference between the groups, chronic infection may not have affected height but may have impacted other aspects of early development, such as nutritional status. If helminthiasis is discovered, early treatment might result in a better prognosis. This is also consistent with the findings of earlier studies conducted in Bandung District. Even though the likelihood of STH infection is quite low, in several areas, prevention efforts can be strengthened. The Worm Eradication Program must still be continued, and the diagnosis of STH is another area that may be improved. The Kato–Katz method can be used to analyze stool samples because it is affordable, simple, widely used, and sensitive in identifying mild and severe STH infections. If a diagnosis of STH infection is confirmed, treatment can begin immediately to prevent chronic infection [16].

Socioeconomic status and environmental variables play a role in the prevalence and occurrence of STH infection. These variables indicate that the father’s education, the water source, and personal hygiene significantly matter [17]. Children’s behavior will be influenced by the parents’ behavior, making the father’s education a determining factor both as a role model and in providing family necessities, such as personal hygiene and clean water [18]. In this study, we discovered that the prevalence was higher when the father’s education was lower (*p* = 0.010) when artificial clean water sources such as an electric pump were used (*p* = 0.020), when the distance between the water source and the toilet was less than 10 m (*p* = 0.022), and when personal cleanliness was lower in the total community. Individual characteristics, particularly toenail cleanliness, were significant (*p* = 0.001).

Additionally, the prevalence of STH infection in children of normal and short stature was determined by both the source of clean water and the distance from the water source to the toilet. These findings are consistent with those of a study conducted on children under the age of five years in Indonesia in 2016, which found that environmental factors such as the condition of clean water supply facilities, feces disposal facilities, and wastewater disposal facilities influenced the prevalence of STH infection. In that study, the authors found that individual personal hygiene, specifically nail cleanliness, was a major predictor of the occurrence of STH infection in stunted children [5]. This finding is also in line with that of Binga et al., 2022. Parasitic diseases were discovered to be the result of contaminated clean water, inadequate sanitation, and poor personal hygiene. According to the study findings, not washing hands before eating increased the chance of STH infection by 4.13 times [19]. In the Dembiya region, Gizaw et al., 2019, found that children who had parasite diseases lived in locations with limited access to sanitation. The authors also looked at the impact of water, sanitation, and personal hygiene education on the prevalence of STH infection. Compared with previously, providing this education had a strong effect on the prevalence of STH infection [20]. This indicates that health education can be used to drive behavioral changes that reduce the frequency of STH infection, particularly in poor countries such as Indonesia. Faridah et al., 2021, conducted a study in Bandung, reporting that over 85% of children have a low–moderate awareness of STH infection prevention measures, symptoms, and therapy [21].

STH infection, especially *Ascaris lumbricoides*, is a widespread parasitic infection that affects humans globally and has been influencing the world’s population for centuries; about one billion people worldwide are infected with it, especially in tropical areas and rural areas [22]. This is caused by the female worm’s ability to produce a large quantity of eggs; they can release up to 200.000 eggs per day that exhibit high resistance to environmental conditions. Additionally, the easy transmission of contamination among individuals may result from the ingestion of eggs [23,24]. Other helminths produce eggs less than *Ascaris lumbricoides*, such as *Trichuris trichiura* and *Necator americanus*, which can only produce up to 10.000 eggs per day [25,26]. This is consistent with our study because our study was also conducted in rural and densely populated areas; if there is a spread of *Ascaris lumbricoides*, which produce a large number of eggs, the spread will become easier. Meanwhile, other worms are less common. In addition, even though the Kato–Katz examination has been performed three times, the small number of eggs may affect the chances of obtaining species other than Ascaris.

Furthermore, there were no significant differences in severity levels based on egg count between the group of normal and stunted children (*p* > 0.05). After entering the small intestine lumen, the larvae mature into adult worms within approximately 20 days, and female worms are capable of producing around 200.000 eggs daily. These eggs transform into an infective form within two to eight weeks and remain viable for up to 17 months [27]. Adult worms can survive up to two years and typically do not induce acute symptoms [28]. This aligns with the absence of significant differences between normal children and stunted children (*p* > 0.05) found in this study; rapid egg production had not progressed into a chronic state, thus avoiding the induction of growth disorders in the short term.

In this study, 25(OH)D, IL-5, and IL-13 levels were measured to identify their relationship with STH infection. We found no significant differences in children of normal stature, so we focused on the stunted group; no correlation was found between 25(OH)D levels and the prevalence of STH infection in the stunted group. Fisher’s exact test was used to determine the cut-off point for 25(OH)D levels and STH infection, revealing a value of 42.202 ng/mL. This indicates that even children with sufficient 25(OH) levels, according to the Endocrine Society classification, are at risk of STH infection.

In this study, the subjects were children who lived in tropical areas with high exposure to sunlight, so the average levels of vitamin D, which is synthesized from sunlight absorbed from the skin that is converted to previtamin D, may have been higher [29]. This may have led to some of the study groups having 25(OH)D levels above 29 ng/ mL, so the cut-off point was quite high, even though the mean 25(OH)D level in the case group was 28.773 ng/mL.

In this study, the relationship between the prevalence of STH infection and IL-5 and IL-13 levels was investigated. IL-5 can influence the activity of other immune cells, such as T, B, and dendritic cells. IL-5 can influence the differentiation and activation of T-helper type 2 (Th2) cells, which play a role in the immune response to allergies and parasitic infections [30]. IL-13 plays roles in stimulating the differentiation of Th2 cells and inhibiting the differentiation of Th1 cells, which are involved in the adaptive immune response to infection, including parasitic infection. This can lead to an immune response that is more focused on humoral and allergic responses [31].

In this study, a significant difference was found between the prevalence of STH infection and the levels of IL-5 (*p* = 0.008; OR = 6.93) and IL-13 (*p* < 0.01; OR = 16.71), meaning that someone with an IL-5 or an IL-13 level below the cut-off point had a 6.93 or 16.71 times higher risk of STH infection, respectively, compared with someone with a higher value. These results can contribute to selecting diagnostic tests to assess the immunological status related to STH, especially in high-risk areas.

## 5. Conclusions

In this study, we found that the prevalence of STH infection was 5.7% in stunted children and 3.4% in normal-height children aged 24–59 months. Environmental factors, namely, parental education, water source, distance between water sources and toilets, and cleanliness of toenails, were found to be key elements for the transmission of STH infection.

We further studied the stunted children within this population, finding an association of IL-5, IL-13, and 25(OH)D levels with the prevalence of STH infection. IL-5, IL-13, and 25(OH)D levels were negative predictors of infection in stunted children, meaning that a child with lower IL-5, IL-13, and 25(OH)D levels would be more vulnerable to becoming ill if infected by STHs. We found a simultaneous relationship between IL-5 and IL-13 levels and environmental factors and the prevalence of STH infection in stunted children.

## 6. Study Limitations

In this study, the 25(OH)D, IL-5, and IL-13 levels were not measured serially after therapy to observe differences in post-therapy levels. Furthermore, other factors such as parental height, secondary infections, genetic disorders, and others that can influence the prevalence of stunting were not examined. The immune response did not differentiate between different species because the species was *Ascaris lumbricoides*.

A low number of STH infections was found, even though the statistical analysis suggests an adequate sample size for infection cases. The low prevalence rates may affect the validation strength of the results.

## Figures and Tables

**Table 1:** Characteristics children aged 24–59 months.

Characteristic		STH Infection		*p* Value *
		Positive (n = 33)	Negative (n = 661)	
1.	Sex			0.491
	Male	19 (57.5%)	340 (51%)	
	Female	14 (42.5%)	321 (49%)	
2.	Body height			0.156
	Stunted	23 (5.7%)	378 (94.3%)	
	Normal	10 (3.4%)	283 (96.6%)	
3.	Mother’s education:			0.138
	Elementary school	12 (9.2%)	119 (90.8%)	
	Junior high school	12 (4.0%)	291 (96.0%)	
	Senior high school	8 (3.4%)	228 (96.6%)	
	Bachelor’s degree	1 (4.2%)	23 (95.8%)	
4.	Father’s education:			<0.001
	Elementary school	15 (12.5%)	105 (87.5%)	
	Junior high school	7 (2.4%)	281 (97.6%)	
	Senior high school	9 (3.5%)	250 (96.5%)	
	Bachelor’s degree	2 (7.4%)	25 (92.6%)	
5.	Nutritional status:			0.674
	Severely underweight	8 (4.3%)	178 (95.7%)	
	Underweight	9 (7.3%)	114 (92.7%)	
	Normal	16 (4.2%)	366 (95.8%)	
	Overweight	0	2 (100%)	
	Obese	0	1 (100%)	
6.	Water sources			0.009
	Tap water	3 (1.8%)	164 (98.2%)	
	Water well	5 (3.5%)	136 (96.5%)	
	Water pump	3 (3.3%)	87 (96.7%)	
	Water electric pump	22 (8.4%)	239 (91.6%)	
	Spring water	0	35 (100%)	
7.	Distance between water sources and toilet			0.002
	Less than 10 m	23 (7.6%)	280 (92.4%)	
	More than 10 m	10 (2.6%)	381 (97.4%)	
8.	Hand washing habit			0.580
	With water only	14 (4.3%)	313 (95.7%)	
	With water and soap	19 (5.2%)	348 (94.8%)	
9.	Cleanliness of toenails			0.013
	Clean	23 (3.9%)	566 (96.1%)	
	Dirty	10 (9.5%)	95 (90.5%)	

* *p* value: chi-square test.

**Table 2:** Comparison of the severity levels, based on number of eggs, of STH infection between normal children and stunted children.

Children Infected by *Ascaris lumbricoides*				
Subject	Species	Number of Eggs (epg)	Severity Levels of STH	Height per Age
Patient 4	*Ascaris lumbricoides*	250 epg	Mild	Normal
Patient 7	*Ascaris lumbricoides*	600 epg	Mild	Normal
Patient 8	*Ascaris lumbricoides*	2.000 epg	Mild	Normal
Patient 9	*Ascaris lumbricoides*	2676 epg	Mild	Normal
Patient 3	*Ascaris lumbricoides*	3.450 epg	Mild	Normal
Patient 5	*Ascaris lumbricoides*	22.600 epg	Moderate	Normal
Patient 6	*Ascaris lumbricoides*	33.450 epg	Moderate	Normal
Patient 2	*Ascaris lumbricoides*	37.250 epg	Moderate	Normal
Patient 1	*Ascaris lumbricoides*	172.250 epg	Severe	Normal
Patient 28	*Ascaris lumbricoides*	59 epg	Mild	Stunted
Patient 25	*Ascaris lumbricoides*	117 epg	Mild	Stunted
Patient 29	*Ascaris lumbricoides*	118 epg	Mild	Stunted
Patient 22	*Ascaris lumbricoides*	147 epg	Mild	Stunted
Patient 27	*Ascaris lumbricoides*	235 epg	Mild	Stunted
Patient 30	*Ascaris lumbricoides*	293 epg	Mild	Stunted
Patient 21	*Ascaris lumbricoides*	513 epg	Mild	Stunted
Patient 17	*Ascaris lumbricoides*	700 epg	Mild	Stunted
Patient 24	*Ascaris lumbricoides*	882 epg	Mild	Stunted
Patient 16	*Ascaris lumbricoides*	1.450 epg	Mild	Stunted
Patient 11	*Ascaris lumbricoides*	1.500 epg	Mild	Stunted
Patient 18	*Ascaris lumbricoides*	2.950 epg	Mild	Stunted
Patient 12	*Ascaris lumbricoides*	3.150 epg	Mild	Stunted
Patient 15	*Ascaris lumbricoides*	3.850 epg	Mild	Stunted
Patient 31	*Ascaris lumbricoides*	4.841 epg	Mild	Stunted
Patient 14	*Ascaris lumbricoides*	6.700 epg	Moderate	Stunted
Patient 26	*Ascaris lumbricoides*	19.450 epg	Moderate	Stunted
Patient 23	*Ascaris lumbricoides*	25.470 epg	Moderate	Stunted
Patient 13	*Ascaris lumbricoides*	28.500 epg	Moderate	Stunted
Patient 19	*Ascaris lumbricoides*	53.350 epg	Severe	Stunted
Patient 10	*Ascaris lumbricoides*	64.500 epg	Severe	Stunted
Patient 20	*Ascaris lumbricoides*	64.550 epg	Severe	Stunted

**Table 3:** Relationship of 25(OH)D, IL-5, and IL-13 levels with STH infection.

Variable	Group				*p* Value
	A (n = 20)	B (n = 20)	C (n = 10)	D (n = 30)	
**25(OH)D** (ng/mL)	28.773 (8.462)	32.757 (12.432)	32.128 (12.038)	30.927 (11.248)	0.701
A vs. B					0.258
A vs. C					0.435
A vs. D					0.501
B vs. C					0.884
B vs. D					0.568
C vs. D					0.767
**IL-5** (pg/mL)	60.886 (21.812–216.271)	89.508 (25.050–199.098)	71.575 (18.624–190.132	81.262 (10.867–309.279)	**0.080**
A vs. B					**0.006**
A vs. C					0.397
A vs. D					0.104
B vs. C					0.183
B vs. D					0.452
C vs. D					0.656
**IL-13** (pg/mL)	5.456 (0.612–76.351)	15.455 (0.853–386.627)	11.839 (1.149–495.728)	11.724 (1.637–222.731)	**0.063**
A vs. B					**0.010**
A vs. C					0.397
A vs. D					**0.022**
B vs. C					0.713
B vs. D					0.362
C vs. D					0.939

Note: Data are presented as means for 25(OH)D and medians and ranges for IL-5 and IL-13. Group A: Stunted children with STH infection. Group B: Stunted children without STH infection. Group C: Normal-height children with STH infection. Group D: Normal-height children without STH infection.

**Table 4:** Characteristics of stunted children aged 24–59 months.

Characteristics		STH Infection		*p* Value
		Positive (n = 20)	Negative (n = 20)	
1.	Sex			0.527
	Male	11	9	
	Female	9	11	
2.	Mother’s education:			0.604
	Elementary school	6	9	
	Junior high school	10	7	
	Senior high school	4	3	
	Bachelor’s degree	0	1	
3.	Father’s Education:			0.187
	Elementary school	10	4	
	Junior high school	5	9	
	Senior high school	5	6	
	Bachelor’s degree	0	1	
4.	Nutritional Status:			0.618
	Severely underweight	9	7	
	Underweight	6	9	
	Normal	5	4	
5.	Water sources			0.020
	Tap water	1	6	
	Water well	4	5	
	Water pump	3	6	
	Water electric pump	12	0	
	Spring water	0	3	
6.	Distance between water sources and toilet			0.011
	Less than 10 me	14	6	
	More than 10 m	6	14	
7.	Hand-washing habits			0.744
	With water only	7	8	
	With water and soap	13	12	
8.	Cleanliness of toenails			0.525
	Clean	12	10	
	Dirty	8	10	

**Table 5:** Cut-off points of 25(OH)D, IL-5, and IL-13 levels as predictors of the prevalence of STH infection in stunted children.

Variable	*Cut-Off Point*	AUROC (95% CI)
25(OH)D (ng/mL)	≤42.202	0.605 (0.438–0.756)
IL-5 (pg/mL)	≤62.774	0.750 (0.588–0.873)
IL-13 (pg/mL)	≤6.708	0.735 (0.572–0.862)

AUROC: area under the ROC; ROC: receiver operating characteristic.

**Table 6:** Relationship of cut-off points of 25(OH)D, IL-5, and IL-13 level with prevalence of STH infection in stunted children.

Variable	STH Infection		*p* Value *	OR (95% CI)
	Negative (n = 20)	Positive (n = 20)		
25(OH)D (ng/mL)			0.020 **	-
≤42.202	20	14		
>42.202	0	6		
IL-5 (pg/mL)			0.008	6.93 (1.53–31.38)
≤62.774	11	3		
>62.774	9	17		
IL-13 (pg/mL)			<0.001	16.71 (2.98–93.88)
≤6.708	13	2		
>6.708	7	19		

* *p* value: chi-square test. ** *p* value: Fisher’s exact test. OR (95% CI): odds ratio (95% confidence interval 95%).

**Table 7:** Correlation of cut-off points of IL-5 and IL-13 levels with the prevalence of STH infection in stunted children based on results of multiple logistic regression.

Variable	Coef B	SE (B)	*p* Value	OR_adj_ (95% CI)
IL-5 (≤62.774)	2.239	1.287	0.041	9.38 (1.13–77.88)
IL-13 (≤6.708)	3.166	1.290	0.007	23.71 (2.84–197.94)
Distance between water sources with toilet (<10 m)	2.380	1.173	1.173	10.81 (1.57–74.40)

Note: Model accuracy: 85%; R^2^ (Nagelkerke): 0.683; OR_adj_: adjusted odds ratio.

## Data Availability

The data presented in this study are available on request from the corresponding author.
